# Circular RNA hsa_circ_0006848 Related to Ribosomal Protein L6 Acts as a Novel Biomarker for Early Gastric Cancer

**DOI:** 10.1155/2019/3863458

**Published:** 2019-09-02

**Authors:** Jun Lu, Peng-yang Zhang, Jian-wei Xie, Jia-bin Wang, Jian-xian Lin, Qi-yue Chen, Long-long Cao, Ping Li, Chao-hui Zheng, Chang-ming Huang

**Affiliations:** ^1^Department of Gastric Surgery, Fujian Medical University Union Hospital, Fuzhou, China; ^2^Department of General Surgery, Fujian Medical University Union Hospital, Fuzhou, China; ^3^Key Laboratory of Ministry of Education of Gastrointestinal Cancer, Fujian Medical University, Fuzhou, China

## Abstract

**Objective:**

Circular RNAs (circRNAs) have been reported to be widely involved in pathological processes of various cancers. However, little is known about their diagnostic values in early gastric cancer (EGC). This study is aimed at exploring whether circulating circRNAs in plasma could act as biomarkers for EGC diagnosis.

**Materials and Methods:**

Mass spectrometry (MS) was performed to identify the proteins that at significantly aberrantly levels in gastric cancer (GC) tissues. The target circRNA was identified by bioinformatics analysis. A receiver operating characteristic (ROC) curve was generated to evaluate the diagnostic utility.

**Results:**

MS revealed that the ribosomal protein L6 (RPL6) expression was significantly downregulated only in EGC tissues vs. nontumorous tissues; this was validated by western blotting (*n* = 30, *p* = 0.0094). Bioinformatics analysis predicted that there is a hsa_circ_0006848/hsa_miR-329-5p/RPL6 axis in GC progression. The hsa_circ_0006848 expression was significantly downregulated in EGC tissues (vs. nontumorous tissues, *n* = 30, *p* = 0.0073) and plasma samples from EGC patients (vs. paired healthy volunteers, *n* = 30, *p* = 0.0089). In addition, the hsa_circ_0006848 plasma level in postoperative patients was significantly higher than that of preoperative patients (*n* = 30, *p* = 0.047). Furthermore, the decreased hsa_circ_0006848 expression in plasma was negatively correlated with poor differentiation (*p* = 0.037) and tumor size (*p* = 0.046). The area under the ROC curve (AUC) of hsa_circ_0006848 in plasma was 0.733, suggesting a good diagnostic value. The plasma hsa_circ_0006848 level combined with the carcinoembryonic antigen (CEA), carbohydrate-associated antigen 19-9 (CA19-9), and carbohydrate-associated antigen 72-4 (CA72-4) level increased the AUC to 0.825.

**Conclusion:**

Our results indicated that plasma hsa_circ_0006848 may be a novel noninvasive biomarker in EGC diagnosis.

## 1. Introduction

Gastric cancer (GC) is one of the major causes of cancer-associated mortality worldwide [1]. The prognosis of GC patients is inversely proportional to the cancer stage when diagnosed, and the early detection of GC is crucial to enhance patients' survival. Early gastric cancer (EGC) was defined as a “carcinoma limited to the gastric mucosa and/or submucosa regardless of the lymph node status” [2]. According to the results of the 2009 annual report of the Japanese Gastric Cancer Association (JGCA) nationwide registry, the 5-year survival rate of advanced gastric cancer is still very poor; however, the 5-year survival rate of patients with EGC after surgical treatment has over 90% [3]. China is a high-risk region for GC [4], while the detection rate of EGC in China only accounts for 5%-20% of total GC [5]. Gastric endoscopy is widely used for the early detection of GC; however, it is worth noting that gastroscopic diagnosis of EGC is difficult and the gastroscopic results depend on the skills and consciousness of the endoscopists, because lesion of EGC is very subtle so that it may be easily missed during gastroscopy [5]. In addition, currently available serum tumor biomarkers, such as carcinoembryonic antigen (CEA), carbohydrate antigen 19-9 (CA19-9), and carbohydrate antigen 72-4 (CA72-4), have the unsatisfactory sensitivity and specificity in gastrointestinal cancers [6, 7]. Therefore, discovering novel biomarkers to improve the detection of EGC is urgently in demand.

circRNAs are a new type of endogenous noncoding RNA (ncRNA), which is produced by posterior grafting and is characterized by a covalent closed-loop free of 3′ and 5′ ends [8]. Due to the closed-loop structure, circRNAs could resist to the digestion induced by exonuclease and more stable in plasma compared to linear RNAs, such as microRNAs (miRNAs), and long noncoding RNAs (lncRNAs). On the other hand, circRNAs often exhibit a tissue-specific and developmental stage-specific expression [9]. These properties offer the potential for circRNAs to be an ideal diagnostic biomarker in cancer. Some previous studies have confirmed that circRNAs can be used as diagnostic biomarkers for gastric cancer [10, 11], but studies on circRNAs and EGC are still rarely reported.

In this study, hsa_circ_0006848 was investigated (http://www.circbase.org/cgi-bin/simplesearch.cgi). Its gene is located at chr1:42730785-42744343; its relative gene symbol is FOXJ3 (forkhead box J3). We first found that plasma hsa_circ_0006848 may be a new kind of potential circulating biomarker for diagnosing EGC.

The reason why hsa_circ_0006848 was selected as the object of this study is that in our previous Mass Spectrometric analysis, ribosomal protein L6 (RPL6), which as early detection marker of cancer [12], was found to be significantly downregulated in EGC, and more interestingly, hsa_circ_0006848 may exert an oncogenic role in GC through sponging miR-329-5p targets RPL.

## 2. Materials and Methods

### 2.1. Clinical Specimens

A total of 46 pairs of gastric cancer tissues and adjacent nontumor tissues were obtained from patients with early gastric cancer at Fujian Medical University Union Hospital between May 2015 and May 2016, 30 pairs of which for RT-PCR and 16 of which for LC-MS/MS (Mass Spectrometric). These tissue specimens were immediately conserved in RNA-fixer Reagent (BioTeke, Beijing, China) after removal from the patients' stomach and were stored at -80°C until use. All patients underwent radical gastrectomy. None of the patients underwent preoperative chemotherapy or radiotherapy. Postoperative adjuvant chemotherapy was performed with 5-fluorouracil-based drugs plus oxaliplatin in advanced cases. Besides, 30 paired plasma samples of preoperative and postoperative from 30 early gastric cancer patients, and the control fasting plasma from 30 healthy volunteers, gender and age matched to patients, were obtained from Fujian Medical University Union Hospital, between March 2018 and May 2018. The peripheral blood samples (5 ml) obtained from preoperative and postoperative patients (10 days after surgery) and controls were collected in BD Vacutainer tubes (BD, New Jersey, USA) and then centrifuged (3000×g for 10 min). Subsequently, the plasma samples were kept at -80°C. The pathologic stage of the tumor was reassessed according to the 2010 International Union Against Cancer (UICC) TNM classification on gastric cancer (seventh edition). This study was approved by the ethics committee of Fujian Medical University Union Hospital, and written consent was obtained from all patients involved.

### 2.2. Mass Spectrometric

Protein bands were excised from the SDS-PAGE gel, destained, and then reduced and blocked in 10 mM TCEP and 40 mM 2-chloroacetamide at 37°C for 30 min. The protein bands were then digested in-gel with sequencing-grade trypsin (10 ng/*μ*l trypsin, 50 mM ammonium bicarbonate, pH 8.0) at 37°C. The peptides were extracted with 100% acetonitrile followed by 5% formic acid/100% acetonitrile and then concentrated to a volume of ∼20 *μ*l. The extracted peptides were separated in an analytical capillary column (75 *μ*m × 30 cm) that was packed with a 1.7 *μ*m spherical C18 reverse phase material (YMC, Kyoto, Japan). A NanoLC-1000 (ThermoFisher) binary pump was used to generate the HPLC gradient as follows: 0-5% B for 4 min, 5-40% B for 20 min, and 40-100% B for 8 min (A = 0.1% acetic acid in water; B = 0.1% acetic acid/90% acetonitrile). The eluted peptides were sprayed into a Q mass spectrometer that was equipped with a nano-ESI ion source. The mass spectrometer was operated in a data-dependent acquisition (DDA) mode with one MS scan followed by three MS/MS scans for each cycle. The data were processed using Proteome Discoverer software (version 2.1, ThermoFisher) with 1% False Discovery Rate (FDR) at both peptide and protein level. Search criteria included carbamidomethylation of cysteine as a fixed modification and oxidation of methionine and acetyl (protein N terminus) as variable modifications.

### 2.3. RNA Isolation and Real-Time Quantitative Polymerase Chain Reaction

RNA isolation was performed using the Trizol reagent according to the manufacturer's manual. Reverse transcription was performed with the ReverTra Ace qPCR RT kit (Toyobo, Osaka, Japan) using random primers and 1 *μ*g of mRNA per reaction as a template. RT-qPCR analysis was performed in triplicate on the ABI Prism 7500 Sequence Detection System (Thermo Fisher Scientific) using the Thunderbird SYBR qPCR Mix (Toyobo) according to the manufacturer's recommendations. The primers' sequences of GAPDH were the control in these experiments. Primer sequences were as follows: hsa_circ_0006848: 5′-TGCCTCGATCTAAGGATGACC-3′ (forward) and 5′-TGAGCTGTGGTAACCAGTCC-3′ (reverse); and GAPDH: 5′-GGTCGGAGTCAACGGATTTG-3′ (forward) and 5′-ATGAGCCCCAGCCTTCTCCAT-3′ (reverse).

### 2.4. Western Blotting Analysis

The methods were described as we previously described [13]. Anti-RPL6 (ab126100) were purchased from Cell Signaling Technology. Anti-GAPDH (ab181602) antibodies were purchased from Abcam. AKT siRNA (#6211) and control siRNA (#6568) were purchased from Cell Signaling Technology. We corrected the loading error based on loading controls and compared the expression levels of target proteins in gastric tumor and adjacent nontumor tissues. The protein expression in tumors was defined as high level when it was higher than that in normal tissue but was defined as low level when it was lower than that in normal tissue.

### 2.5. Statistical Analysis

Differences of hsa_circ_0006848 levels between GC tissues and paired adjacent nontumor tissues and between plasma samples from GC patients and healthy controls were analyzed using Student's *t*-test. The correlation between hsa_circ_0006848 expression levels and clinicopathological factors was analyzed by one-way analysis of variance (ANOVA) [14]. The receiver operating characteristic (ROC) curve was established to evaluate the diagnostic value. The area under the curve (AUC) ≤ 0.5 represented no diagnostic value. *p* < 0.05 was considered to be statistically significant, and all *p* values were two-sided. All statistical analyses were performed using SPSS software 22.0 (SPSS Inc., Chicago, IL, USA) and GraphPad Prism 5.0 (GraphPad Software, La Jolla, CA, USA).

## 3. Results

### 3.1. Proteins Identified in T1 Stage or Non-T1 Stage (T2-T4 Stage) in Gastric Cancer Samples and Ingenuity Pathway Analysis (IPA)

**Figure 1:**
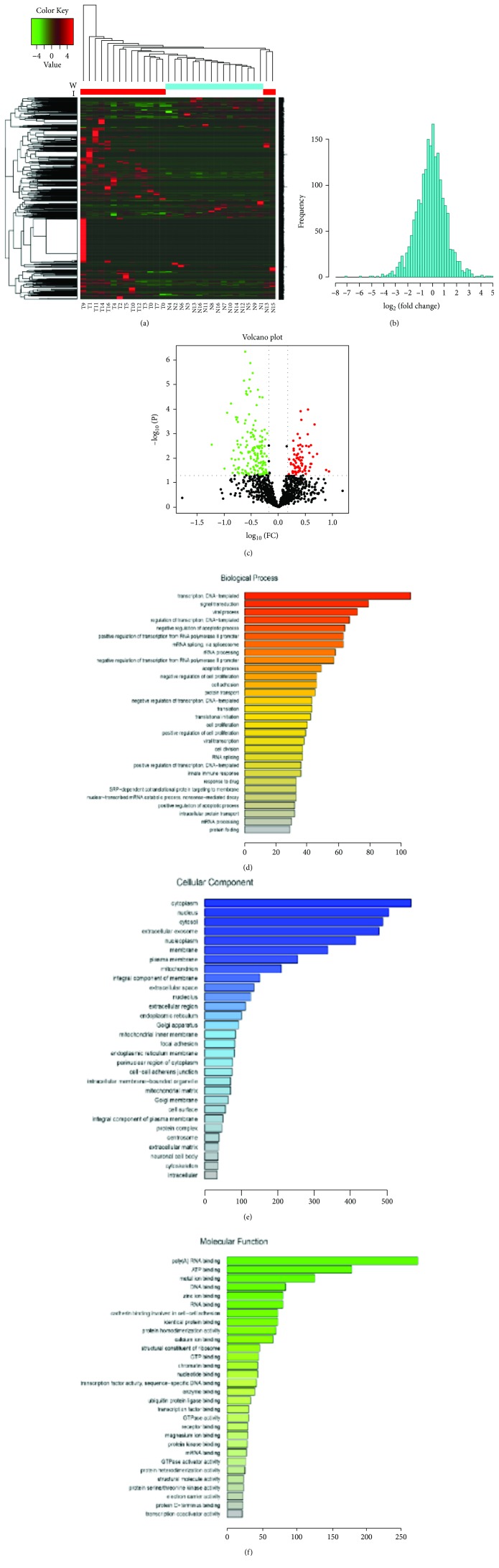
Proteins identified in gastric cancer and Ingenuity Pathway Analysis (IPA). (a) The heat map of proteins which were FDR ≥ 1.5 and *p* < 0.05 (T1-T4 and N1-N4 were T1 stage gastric cancer and corresponding adjacent nonneoplastic tissues; T5-T16 and N5-N16 were non-T1 stage gastric cancer and corresponding adjacent nonneoplastic tissues). (b) The frequency diagram of proteins which were FDR ≥ 1.5 and *p* < 0.05; (c) the volcano plots of proteins which were FDR ≥ 1.5 and *p* < 0.05; (d) Gene Ontology analysis of those proteins in biological process; (e) Gene Ontology analysis of those proteins in cellular component; (f) Gene Ontology analysis of those proteins in molecular function.

We analyzed different stages of gastric cancer samples by LC-MS/MS. Totally, 1308 proteins were identified (fold change > 1.5, *p* < 0.05) (Figures 1(a)–1(c)). Gene Ontology analysis showed that proteins are involved in many biological processes, such as transcription, DNA-templated, signal transduction, viral process, regulation of transcription, DNA-templated, and negative regulation of apoptotic process (Figure 1(d)). Also, proteins are involved in cellular component, such as cytoplasm, nucleus, cytosol, extracellular exosome, and nucleoplasm (Figure 1(e)). Proteins participate in many molecular function including poly(A) RNA binding, ATP binding, metal ion binding, DNA binding, and zinc ion binding (Figure 1(f)).

**Figure 2:**
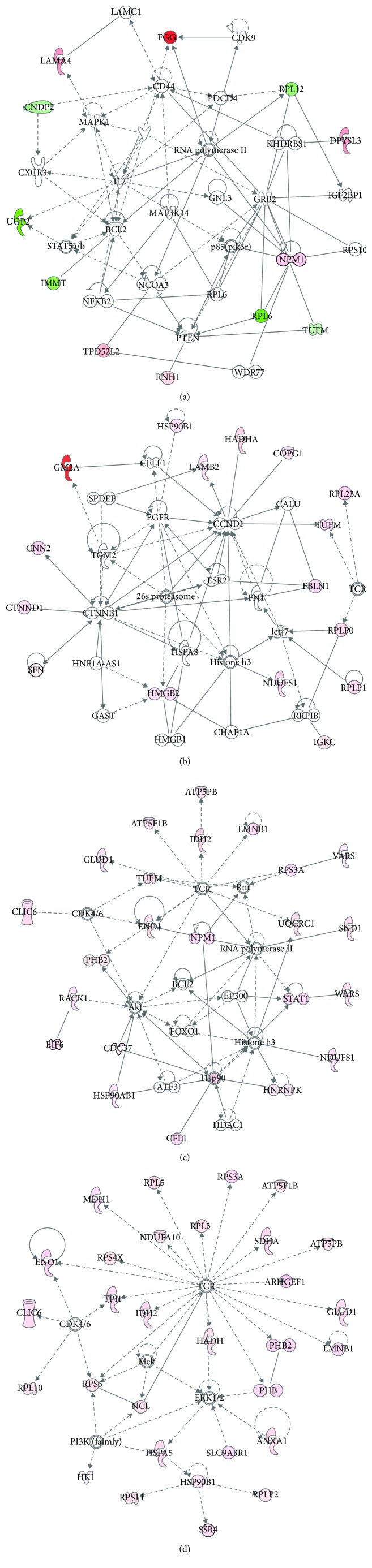
Significant gene networks of different genes in (a) T1, (b) T2, (c) T3, and (d) T4 stage, respectively.

The samples were divided into four groups including the groups of T1 stage, T2 stage, T3 stage, and T4 stage. Figures 2(a)–2(d) showed the significant protein interaction networks of different protein in each stage. As what was mentioned above, EGC has a significant increase in cure rate, so we focused on T1 stage. [Supplementary-material supplementary-material-1] showed that top functions of these genes in T1 stage were related to cardiotoxicity and hepatotoxicity. [Supplementary-material supplementary-material-1] showed that top networks was cancer, organismal injury and abnormalities, and protein synthesis. [Supplementary-material supplementary-material-1] showed that the downregulated molecules include RPL6, UGP2, IMMT, RPL12, CNDP2, and TUFM. RPL6 was the most downregulated protein, so we focused on it.

### 3.2. RPL6 Was Downregulated in Human T1 Stage Gastric Cancer Specimen and Related to hsa_circ_0006848

**Figure 3:**
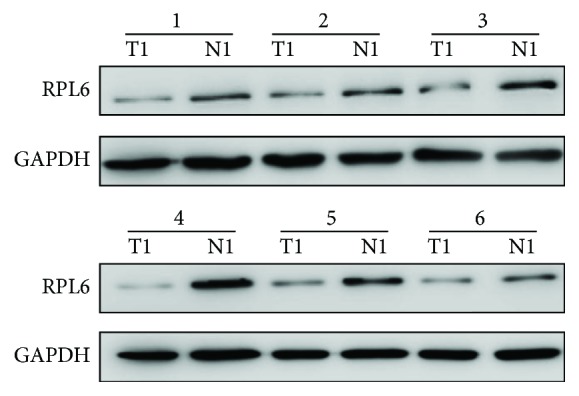
The protein expression of RPL6 was detected by western blot. RPL6 was downregulated in EGC than adjacent nonneoplastic tissues. N: adjacent nontumorous tissue; T: early gastric cancer tissue.

**Figure 4:**
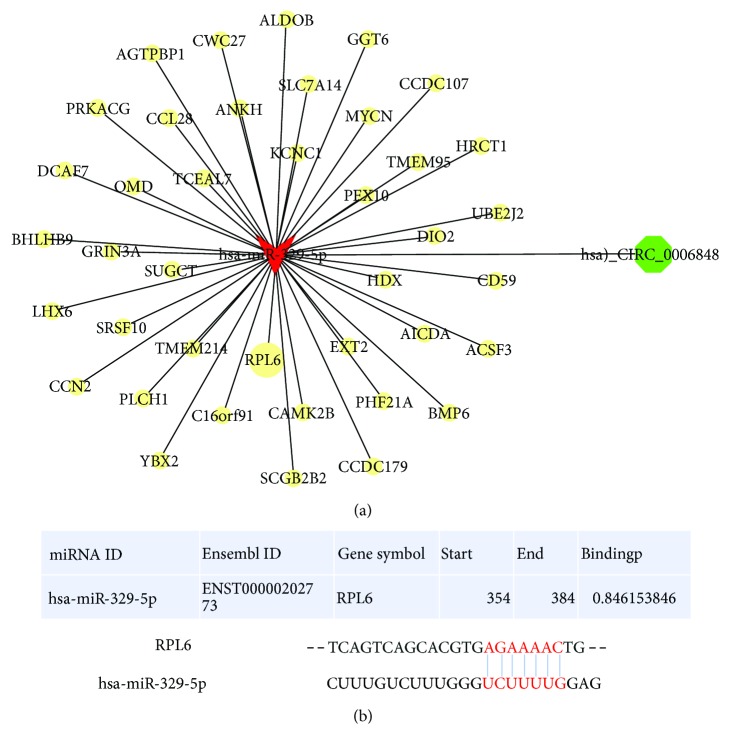
(a) Cytoscape was used to visualize hsa_circ_0006848-hsa_miR-329-5p-RPL6. (b) The binding sites.

In 30 paired EGC and their normal tissues, the expression of RPL6 was dramatically lower in cancer than in the normal tissues (*p* = 0.0094). The result of 6 paired representative samples is shown in Figure 3. Thus, these data suggested that RPL6 was a potential good biomarker in T1 stage gastric cancer. Nowadays, numerous biomarker detection methods were developed, while blood-based biomarkers have attracted tremendous attentions, since they are noninvasive and reliable. Recently, several circRNAs have also been shown to exist in blood and could be used as a new biomarker for cancer diagnosis, because of its high stability, high abundance, and tissue specificity. Therefore, we tried to find a circRNA related to RPL6 and instead of RPL6 to be a new biomarker in T1 stage biomarker. As we all know, the miRNA “sponge” is the most well-known regulatory mechanism for circRNA effects. Thus, we tried to find a target circRNA through miRNA. We predicted the miRNA which may combine with RPL6 through miRWalk (http://mirwalk.umm.uni-heidelberg.de). We found that hsa_miR-329-5p was one of them. We predicted the circRNA-miRNA-mRNA network using Arraystar's homemade miRNA target prediction software and TargetScan and miRanda. Then, we found hsa_miR-329-5p was the top related miRNA.  Figure 4 shows that RPL6 may be the target gene of hsa_miR-329-5p through the control of hsa_circ_0006848.

### 3.3. hsa_circ_0006848 Expression Was Downregulated in EGC Tissues and Plasma Samples of EGC Patients

**Figure 5:**
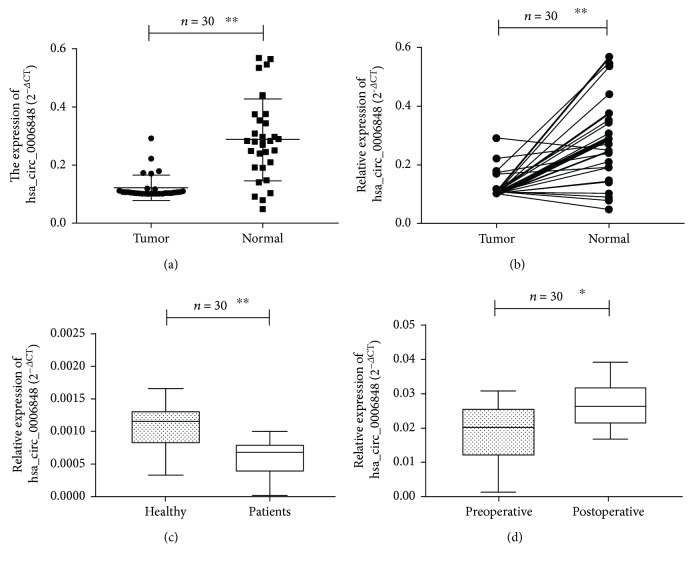
hsa_circ_0006848 was downregulated in EGC patients. (a) hsa_circ_0006848 was downregulated in EGC tissues; (b) hsa_circ_0006848 expression levels in 30 paired EGC tissues and corresponding normal tissues. hsa_circ_0006848 was downregulated in 83.3% (25/30) EGC tissues; (c) hsa_circ_0006848 has lower expression in plasma of EGC patients than the healthy; (d) hsa_circ_0006848 was reduced in preoperative EGC patients. The expression was shown as the 2^-*ΔΔ*Ct^ value.

We tested hsa_circ_0006848 levels in EGC tissues and plasma samples of patients with EGC. As shown in Figure 5(a), we found that the hsa_circ_0006848 expression was downregulated in EGC tissues (*p* = 0.0073). The exact value of the relative expression of has-circ-0006848 in EGC tissues and adjacent nontumor tissue has been shown in Figure 5(b) (*p* = 0.0065) and [Supplementary-material supplementary-material-1]. Furthermore, hsa_circ_0006848 levels in plasma samples of patients with EGC were lower than those in healthy controls (*p* = 0.0089, Figure 5(c)).

### 3.4. Compare hsa_circ_0006848 Plasma Level on Preoperative and Postoperative EGC Patients

We selected 30 cases of EGC patients and detected the plasma levels of hsa_circ_0006848 at preoperative and postoperative stage. The plasma levels of hsa_circ_0006848 in postoperative patients were significantly increased compared to preoperative patients (*p* = 0.047) (Figure 5(d)).

### 3.5. Potential Diagnostic Values of hsa_circ_0006848 in EGC

Since we found that hsa_circ_0006848 expression levels were lower in EGC tissues as well as in plasma, we further analyzed its association with clinicopathological features of patients with EGC. As shown in Table 1, hsa_circ_0006848 levels in plasma from EGC patients were significantly related to tumor differentiation and tumor size (*p* = 0.037 and *p* = 0.046, respectively).

**Figure 6:**
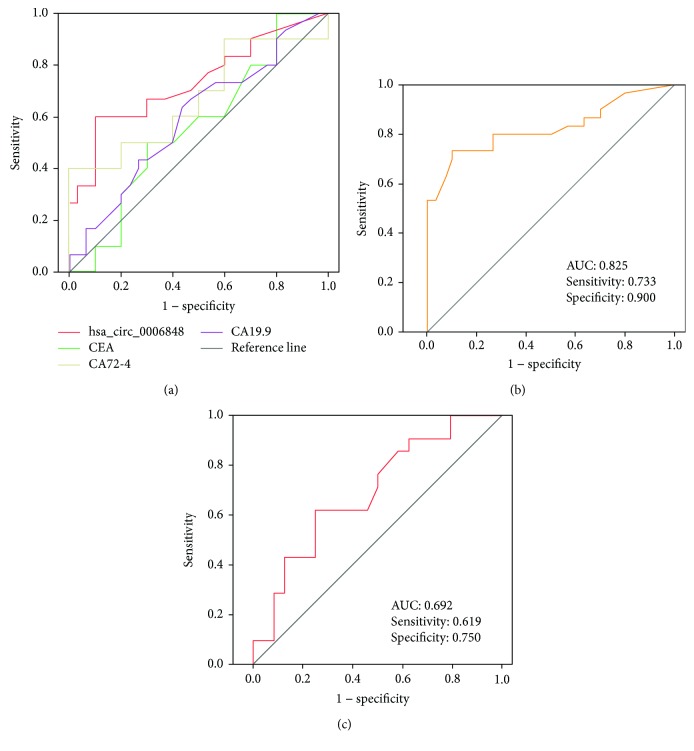
The potential diagnostic values of hsa_circ_0006848. (a) Receiver operating characteristic curve of plasma hsa_circ_0006848, CEA, CA19-9, and CA72-4 levels. (b) Receiver operating characteristic curve of combination of plasma hsa_circ_0006848, CEA, CA19-9, and CA72-4 levels. (c) Receiver operating characteristic curve of plasma hsa_circ_0006848 in EGC with normal CEA, CA19-9, and CA72-4.

To evaluate the potential diagnostic value, a ROC curve was generated for hsa_circ_0006848 levels in plasma. We found that the area under the ROC curve (AUC) was 0.733 (Figure 6(a)). When the expression level of plasma hsa_circ_0006848 was combined with the CEA, CA19-9, and CA72-4 level, the AUC increased to 0.825 (Figure 6(b)).

In the present study, the positive rate of CEA, CA19-9, and CA72-4 was 3.3% (1/30), 3.3% (1/30), and 6.7% (2/30), respectively. Of the 30 EGC patients, 26 patient CEA, CA19-9, and CA 72-4 are all in a normal level. Interestingly, the area under the ROC curve (AUC) of hsa_circ_0006848 in plasma of EGC patients with normal CEA, CA19-9, and CA72-4 level was 0.692 suggesting a good diagnostic value, which further confirm the superiority of the diagnostic value of hsa_circ_0006848 (Figure 6(c)).

## 4. Discussion

The poor prognosis of gastric cancer (GC) is usually due to delayed diagnosis. Gastroscopy is a standard screening tool for gastric lesions; however, its results may be mainly related to the endoscopist's skill, awareness, and ability to identify EGC. Not to mention, patients consider it so uncomfortable. Previous study showed that the detection rate of EGC by intensive gastroscopy was 3.3%, whereas the detection rate of EGC only by white light endoscopy was 0.5% [15]. Another Japanese research suggested that the targeted biopsy under narrow-band imaging (NBI) on doing magnifying gastroscopy for the high-risk patients could enhance the detection of EGC [16]. However, it is impractical to make intensive gastroscopy for each patient in China, where there are a large number of population and poor economic condition. Therefore, it is crucial to identify high-risk patients from whole asymptomatic ones.

It has been proposed that many ribosomal proteins (RPs) may act as cancer genes in human [3]. Recently, more and more RPs were found dysregulated in tumors, such as overexpression of RPL15 in esophageal cancer [17] and high-expression of RPL6 in human gastric cancer cell [18]. More importantly, RPs can act as a marker of early uterine cervix carcinoma [12]. In this study, we first found that RPL6 was downregulated in EGC, which indicated that RPL6 may be a novel marker for EGC. However, peripheral blood markers are more suitable in clinical practice; therefore, we further explore the plasma markers related to RPL6. We thus selected plasma hsa_circ_0006848 for investigation based on preliminary Mass Spectrometric analysis and prediction of its binding to miR-329-5p/RPL6 axis with known relations to GC.

Up to date, a series of studies have explored the diagnostic value of various plasma tumor markers for gastric cancer [7, 11, 15]. Feng et al. investigated 587 EGC patients underwent R0 gastrectomy from a single center and found that the positive rates of CEA, CA19-9, APF, and CA125 were relatively low for EGC (4.3%, 4.8%, 1.5%, and 1.9%, respectively) [15]. Moreover, circulating miRNAs and long noncoding RNAs (lncRNAs) have been investigated for early diagnosis in several cancers, such as breast cancer [19], bladder cancer [20], colorectal cancer [21], and gastric cancer [7, 22].

Nowadays, circRNA is one of the newest types of noncoding RNAs. There are increasing evidences that circRNAs are involved in the development and progression of diseases, especially cancer. Recently, more attention was focused on the clinical cancer diagnostic value of circRNAs [23, 24]. Zhao et al. [23] found that hsa_circRNA_404833, hsa_circRNA_406483, hsa_circRNA_00641, hsa_circRNA_401977, and hsa_circRNA_001640 are aberrantly expressed in early stage lung adenocarcinoma, which might offer potential targets for the future diagnosis of early lung adenocarcinoma. However, the diagnostic value of plasma circRNA for EGC has not been investigated yet.

In the present study, we calculated and analyzed the relevance of clinicopathological parameters and plasma hsa_circ_0006848 expression level in 30 EGC patients, indicating the close relation with histological grade and tumor size. Besides, comparing with CEA (0.529), CA19-9 (0.562), and CA72-4 (0.594), hsa_circ_0006848 had higher AUC value (0.733). Moreover, the combination of hsa_circ_0006848, CEA, CA19-9, and CA72-4 showed the highest AUC value of 0.825. However, the positive rates of CEA, CA19-9, and other regular tumor markers were relatively low for early gastric cancer [15]. On the other hand, the standard serum markers such as CEA and CA19-9 can be detected in patients with alternative types of carcinoma; thus, it exhibits low specificity and sensitivity [25]. Therefore, it is urgent to seek a novel diagnostic biomarker for EGC patients with normal CEA, CA19-9, and CA 72-4. Thus, to further validate the diagnostic efficiency, we investigated the AUC value (0.692) of hsa_circ_0006848 in patients with CEA, CA19-9, and CA72-4 all negative. In further analysis, we found that the plasma level of hsa_circ_0006848 in postoperative patients (0.283 ± 0.043) significantly decreased compared to preoperative patients (0.109 ± 0.037, *p* < 0.01). This phenomenon may be mainly due to the reduction in the release of tumor-derived nucleic acid after tumor resection [26].

There are some limitations in our study. Firstly, it is the retrospective study with a relative small sample. Thus, it would be better if initial or external validation. Secondly, we did not assess the predictive value of postoperative hsa_circ_0006848 on EGC recurrence patterns and prognosis. Thirdly, the molecular mechanisms of how hsa_circ_0006848 works are still largely unknown.

## 5. Conclusion

In conclusion, our data suggest that the expression of RPL6 and its relative hsa_circ_0006848 was significantly downregulated in patients with EGC compared with that in healthy subjects, and hsa_circ_0006848 in plasma may be served as a promising diagnostic biomarker for EGC.

## Figures and Tables

**Table 1:** Relationship of hsa_circ_0006848 expression levels (2^-ΔΔCt^) in plasma with clinicopathological factors of patients with early gastric cancer.

Characteristics	*n* = 30	Plasma hsa_circ_0006848	*p* value
		Mean ± SD	
Age (years)			0.273
<60	13	0.007467 ± 0.000331	
≥60	17	0.023566 ± 0.000716	
Gender			0.421
Male	19	0.006481 ± 0.000523	
Female	11	0.01271 ± 0.00056	
Diameter (cm)			0.046
<2	18	0.013448 ± 0.000824	
≥2	12	0.013225 ± 0.000645	
Histologic type			0.037
Differentiated	22	0.004192 ± 0.000588	
Undifferentiated	8	0.03836 ± 0.00056	
Lymphatic metastasis			0.241
N0	16	0.007399 ± 0.000851	
N1-2	14	0.001814 ± 0.000593	
TNM stage			0.133
I	25	0.00629 ± 0.000613	
II	5	0.005199 ± 0.000479	
CEA (ng/ml)			0.368
<5.0	20	0.004089 ± 0.00052	
≥5.0	10	0.009001 ± 0.000687	
CA19-9 (U/ml)			0.141
<37	23	0.006524 ± 0.000629	
≥37	7	0.007577 ± 0.000703	
CA72-4 (U/ml)			0.212
<6.9	19	0.003638 ± 0.000871	
≥6.9	11	0.007521 ± 0.000863	

## Data Availability

The data used to support the findings of this study are included within the article.

## Supplementary Materials

Supplementary Materials Supplementary 1: Table S1: top functions in T1 stage gastric cancer tissues. Supplementary 2: Table S2: top networks in T1 stage gastric cancer tissues. Supplementary 3: Table S3: top analysis-ready molecules in T1 stage gastric cancer tissues. Supplementary 4: Table S4: the exact value of the relative expression of has-circ-0006848 in EGC tissues and adjacent nontumor tissue.
