# Supplementary material for: Circular RNA hsa_circ_0006848 Related to Ribosomal Protein L6 Acts as a Novel Biomarker for Early Gastric Cancer
Source: Dis Markers. 2019 Sep 2;2019:3863458. doi: 10.1155/2019/3863458 (PMC6746163; doi:10.1155/2019/3863458)
Supplement: Supplementary Materials — Supplementary 1: Table S1: top functions in T1 stage gastric cancer tissues. Supplementary 2: Table S2: top networks in T1 stage gastric cancer tissues. Supplementary 3: Table S3: top analysis-ready molecules in T1 stage gastric cancer tissues. Supplementary 4: Table S4: the exact value of the relative expression of has-circ-0006848 in EGC tissues and adjacent nontumor tissue. [file 3863458.f1.pdf]

Table S1 Top functions in T1 stage gastric cancer tissues

Top Tox Functions		
Cardiotoxicity		
Name	*p*-value	#Molecules
Cardiac Dilation	4.19E-02 – 6.10E-04	1
Cardiac Arrythmia	1.63E-02 – 1.83E-03	1
Tachycardia	9.11E-03 – 9.11E-03	1
Hepatotoxicity		
Name	*p*-value	#Molecules
Liver Hyperplasia/Hyperproliferation	1.65E-01 – 1.32E-02	9
Liver Cholestasis	3.54E-02 – 3.54E-02	1
Hepatocellular Carcinoma	1.34E-01 – 1.34E-01	2

Table S2 Top networks in in T1 stage gastric cancer tissues

Top Networks		
ID	Associated Network Functions	Score
1	Cancer, Organismal Injury and Abnormalities, Protein Synthesis	32

Table S3 Top analysis-ready molecules in T1 stage gastric cancer tissues

Expr Fold Change up-regulated	
Molecules	Expr. Value
FGG	↑13.800
LAMA4	↑ 5.371
TPD52L2	↑ 4.473
DPYSL3	↑ 4.123
RNH1	↑ 2.839
NPM1	↑ 2.308
Expr Fold Change down-regulated	
Molecules	Expr. Value
RPL6	↓ -9.527
UGP2	↓ -7.118
IMMT	↓ -6.107
RPL12	↓ -4.493
CNDP2	↓ -3.804
TUFM	↓ -3.046

Table S4 The exact value of relative expression of has-circ-0006848 in EGC tissues and adjacent non-tumor tissue

Number	Tumor(2^-Δct)	Normal(2^-Δct)	Up/down
1	0.16944083	0.192109398	down
2	0.10283979	0.248273124	down
3	0.105262631	0.102948877	up
4	0.22174447	0.27	down
5	0.29103754	0.25	up
6	0.106003419	0.376311687	down
7	0.100798732	0.307786103	down
8	0.11573368	0.3536	down
9	0.100306888	0.535886731	down
10	0.1786684	0.547146851	down
11	0.106087223	0.297301779	down
12	0.1000021	0.28	down
13	0.1053804	0.090873282	up
14	0.102524127	0.297301779	down
15	0.103086099	0.139660892	down
16	0.17238969	0.24	down
17	0.102506691	0.566441943	down
18	0.107546378	0.21	down
19	0.100333506	0.375541818	down

20	0.100100536	0.147107024	down
21	0.100844275	0.283220971	down
22	0.100874048	0.29	down
23	0.100793215	0.0476956	up
24	0.11809742	0.283220971	down
25	0.10191293	0.343885455	down
26	0.103129179	0.190782401	down
27	0.104910208	0.441351498	down
28	0.111048543	0.570381858	down
29	0.109485898	0.078020659	up
30	0.10579892	0.244855074	down
